# Respiratory symptoms as initial manifestations of interstitial lung disease in clinically amyopathic juvenile dermatomyositis: a case report with literature review

**DOI:** 10.1186/s12887-021-02958-9

**Published:** 2021-11-03

**Authors:** Jingyi Xia, Gaoli Jiang, Tingting Jin, Quanli Shen, Yangyang Ma, Libo Wang, Liling Qian

**Affiliations:** 1 Division of Pulmonary Medicine, Children’s Hospital of Fudan University, 399 Wan Yuan Road, Shanghai, 201102 People’s Republic of China; 2 Department of Radiology, Children’s Hospital of Fudan University, Shanghai, China; 3 Department of Pathology, Children’s Hospital of Fudan University, Shanghai, China

**Keywords:** Interstitial lung disease, Clinically amyopathic juvenile dermatomyositis, Treatment, Mortality

## Abstract

**Background:**

Clinically amyopathic juvenile dermatomyositis (CAJDM) is a clinical subgroup of juvenile dermatomyositis (JDM), characterized by JDM rashes with little or no clinically evident muscle weakness. Interstitial lung disease (ILD) is an uncommon but potentially fatal complication of juvenile dermatomyositis (JDM). While adults with dermatomyositis-associated ILD usually present respiratory symptoms before or at the same time as skin muscle manifestations, only a few studies have covered the onset of respiratory symptoms of ILD in JDM patients, especially CAJDM. There is currently no clear effective treatment regime or any prognostic factors for CAJDM-associated ILD.

**Case presentation:**

Here, we report the first case of a CAJDM patient who presented with respiratory symptoms as the initial manifestation. A 10-year-old male patient presented to the hospital with a complaint of progressive cough and chest pain. Violaceous macule and papules appeared a few days later and he was positive for anti-Ro-52 antibodies. Imaging showed diffuse interstitial infiltration in both lungs and lung function tests showed restrictive and obstructive ventilatory dysfunction. Muscular abnormalities were excluded by thigh magnetic resonance imaging (MRI) and electromyography. Skin biopsy showed pathognomonic findings consistent with DM. Lung biopsy indicated chronic inflammation of the mucosa. This patient was finally diagnosed with CAJDM complicated by ILD and prescribed methylprednisolone, immunoglobulin, prednisolone and mycophenolate mofetil (MMF) for treatment. The patient’s cutaneous and respiratory manifestations were largely improved. We retrospectively reviewed this and another six cases with CAJDM-associated ILD reported previously to better understand its clinical characteristics and effective management.

**Conclusions:**

Initial respiratory symptoms with rapid progression in patients presenting Gottron papules should be considered manifestations of CAJDM-associated ILD. We also found a combination of corticosteroids, IVIG and MMF to be an effective method of arresting the progress of CAJDM-associated ILD and improving the prognosis of the patients.

## Background

Dermatomyositis (DM) presenting during childhood or adolescence, namely juvenile dermatomyositis (JDM), is an uncommon inflammatory disease classically involving cutaneous changes, proximal muscle weakness, and laboratory evidence of myositis. However, unlike adult dermatomyositis in which patients may develop myositis and muscle weakness without cutaneous changes [1], children and adolescents often display the classical cutaneous manifestations of DM for long periods without clinically significant muscle disease [2]. This subset of dermatomyositis in children and adolescents is called clinically amyopathic juvenile dermatomyositis (CAJDM). It is a subtype of idiopathic inflammatory myopathy (IIM) characterized by cutaneous lesions of dermatomyositis (DM) but no or mild muscle involvement in children and adolescents. CAJDM can be easily misdiagnosed because of its atypical JDM manifestations. The prognosis of CAJDM is largely determined by underlying complications. Interstitial lung disease (ILD), a poor outcome predictor in Asian CADM patients, can progress rapidly and cause fatal respiratory failure [3]. If the diagnosis is delayed, ILD may develop rapidly and can lead to an unfavorable outcome. The mortality rate in CADM-associated ILD reached 33% [4], and it was even higher for CAJDM-associated ILD. Patients with CAJDM-associated ILD are often resistant to high-dose glucocorticoids and immunosuppressive treatments [5]. Given that CAJDM-associated ILD is rare, its clinical features are not well understood and effective clinical management remains unclear.

In this article, we describe a new case of CAJDM for which respiratory symptoms were the initial manifestation. We also reviewed seven patients with CAJDM-associated ILD to assess its clinical manifestations, diagnosis, treatments, and prognosis.

## Case presentation

**Fig. 1:**
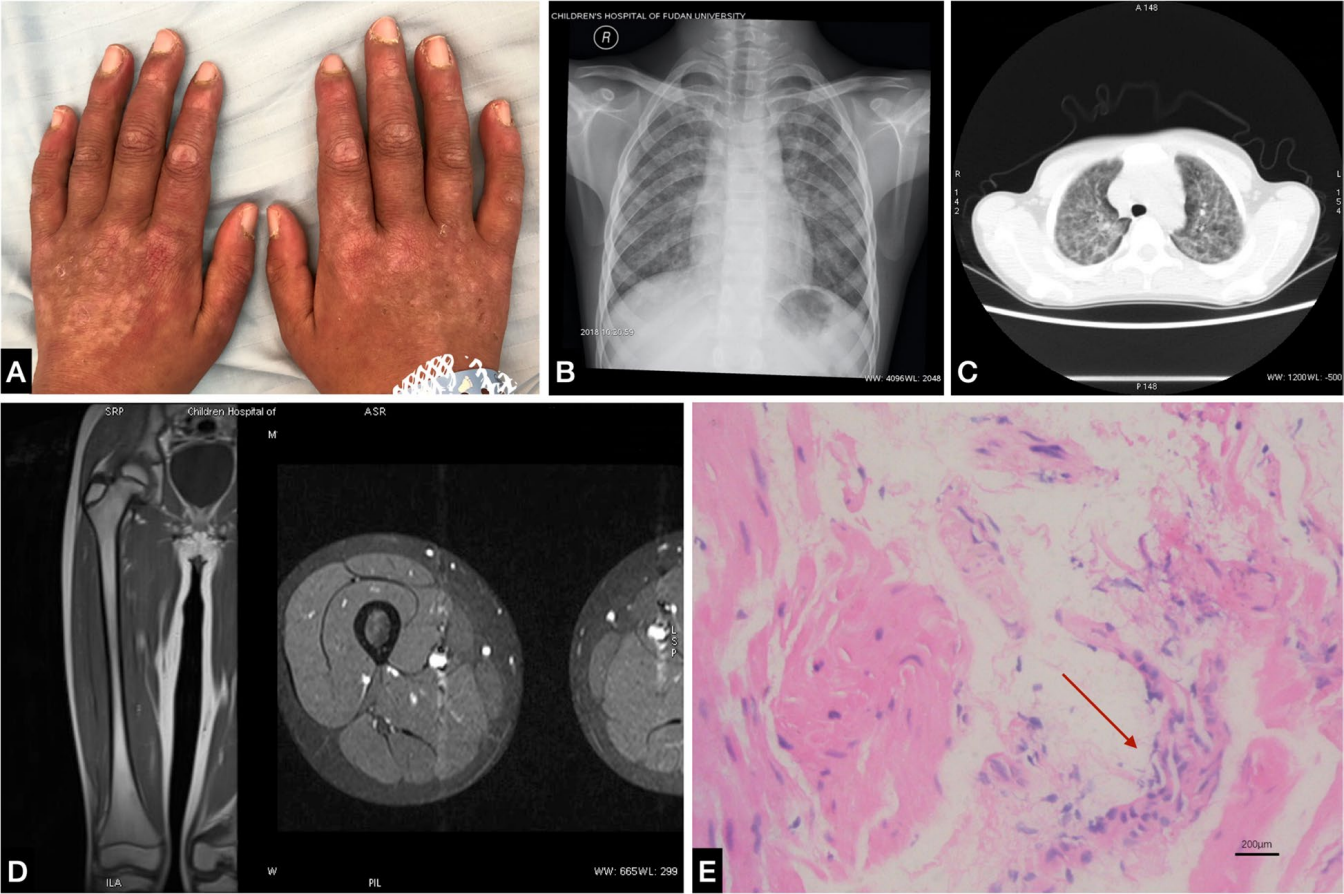
Gottron’s sign, radiological findings and pathological biopsy of CAJDM-ILD. **A**: Typical Gottron papules are present over the knuckles; **B**: Chest radiograph shows diffused interstitial infiltration in both lungs; **C**: Chest HRCT shows diffused interstitial lung changes as grid sign with fibrous streak shadow and right pleural reaction; **D**: Enhancement MRI of the thigh muscle shows no obvious abnormalities; **E**: Pathological biopsy of the basal ridge of the right lung under a bronchoscope shows chronic inflammation of the mucosa with epithelial cells and more neutrophil infiltration (Leica DM750, Leica Application Suite, 2560 × 1920 Full Frame HQ, original magnification× 200)

The patient, a 10-year-old Chinese boy, complained of coughing with chest pain for one month. He began to cough up white sputum and presented with a high fever lasting 3 days with a highest temperature of 39 °C. Later, violaceous macule and papules appeared on his neck, knuckles, and the metacarpophalangeal joints on the back of both hands (Fig. 1A). His respiratory symptoms gradually worsened. His initial oxygen saturation was only 92%, and he did not respond to anti-infective treatments.

The chest radiograph and computed tomography (CT) scan showed diffused interstitial lung disease in both lungs (Fig. 1B-C). The lung function test reported severe restriction and obstruction ventilatory dysfunction: vital capacity (VC), forced vital capacity (FVC) and forced expiratory flow in 1 s (FEV1) were 30% of predicted values (Fig. 3A). All of the breath parameters were measured using the MasterScreen PAED system. The patient was placed in the correct posture, and he was given a nose clip and closed lips around the mouthpiece. He breathed normally and inspired completely and rapidly with a pause of < 2 s. He expired with maximal effort until no more air could be expelled while maintaining an upright posture. Then he was required to inspire with maximal effort until full. He was asked to repeat the instructions three times [6].

His childhood myositis assessment scale (CMAS) score was normal (50/52) and there was no clinical evidence of myositis, muscle weakness, or myalgia. Magnetic resonance imaging (MRI) of thighs, including T2 weighted image(T2WI) and short TI inversion recovery (STIR), also showed normal tissues without any muscle edema or myositis (Fig. 1D). The electrocardiogram and echocardiogram revealed no heart muscle abnormalities.

Laboratory tests showed no elevation of inflammatory markers, including C reactive protein and procalcitonin. Although his serum creatine kinase (CK) level was normal (36 IU/L), his lactic dehydrogenase (LDH) slightly increased to 425 IU/L. Myositis antibody of anti-Ro-52 was positive, whereas anti-Jo-1 and anti-melanoma differentiation-associated protein-5 (anti-MDA-5) and other myositis specific autoantibodies (MSAs) were negative. Anti-cyclic citrullinated peptide antibody, tumor markers, G/GM test, mycoplasma pneumonia antibody (MP-Ab), and sputum culture were all negative.

The liquid-based pathology of the bronchoalveolar lavage fluid (BALF) showed neutrophils (15–20/HP), histiocytes, and epithelial cells. The pathological biopsy of the basal ridge of the right lung under a bronchoscope indicated chronic inflammation of the mucosa (Fig. 1E), and special staining as a periodic acid-Schiff stain (PAS), acid-fast stain, immunohistochemistry, and fungal immunofluorescence of BALF and lung tissue were all negative.

The skin biopsy specimen showed typical findings of DM.

**Fig. 2:**
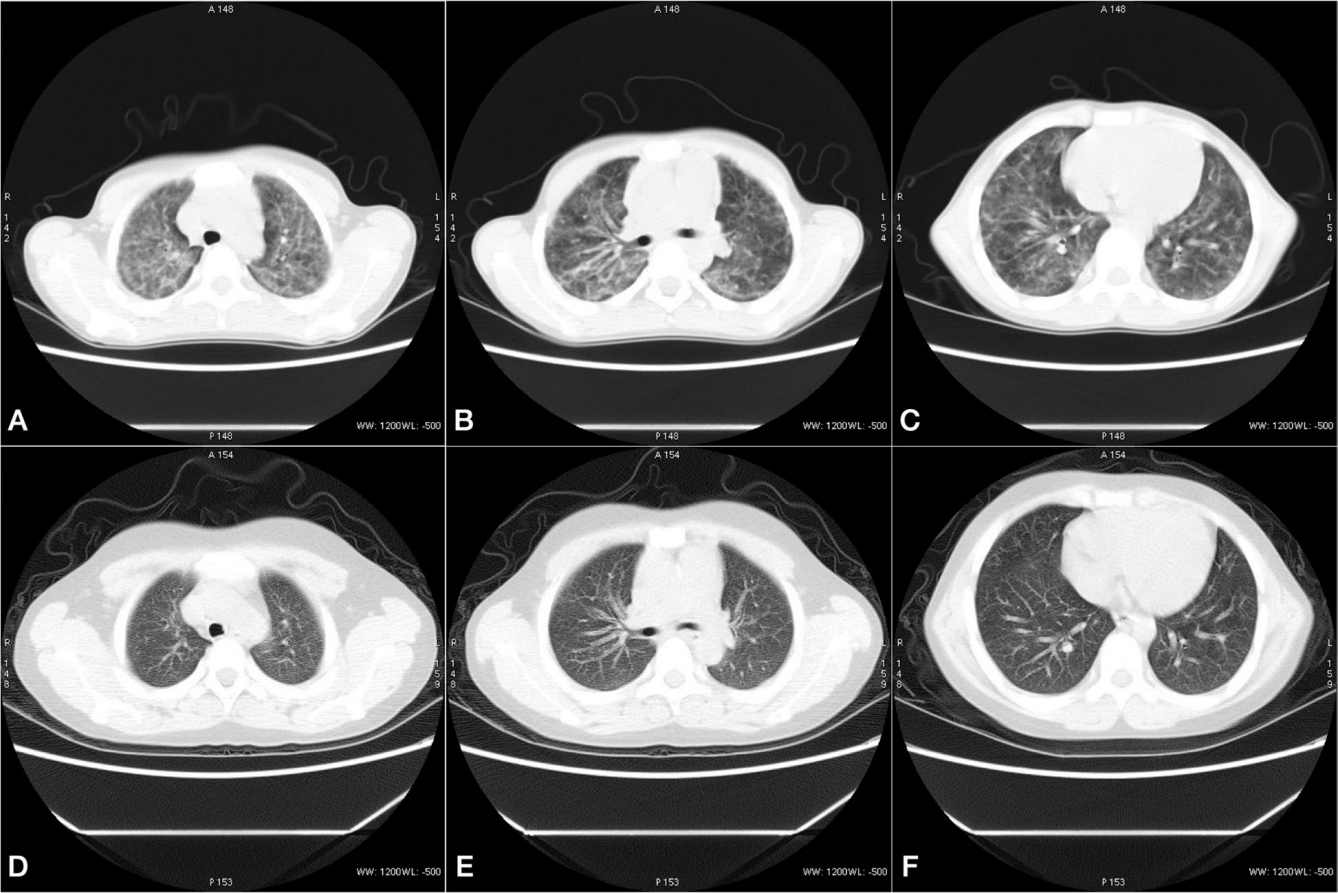
Comparison of chest HRCT images before (Nov. 09, 2018, **A**-**C**) and after (Jan.10, 2019, **D**-**F**) treatments for CAJDM-ILD. A-C shows diffused interstitial changes in both lungs, extensive ground glass shadows, fiber strips with grid syndrome, small mediastinal lymph nodes and partial pleural reaction on the right. **D**-**F** shows multiple clockwork shadows in both lungs, several tiny nodules can be seen in the right lung, and the upper left lung nodule has been absorbed

**Fig. 3:**
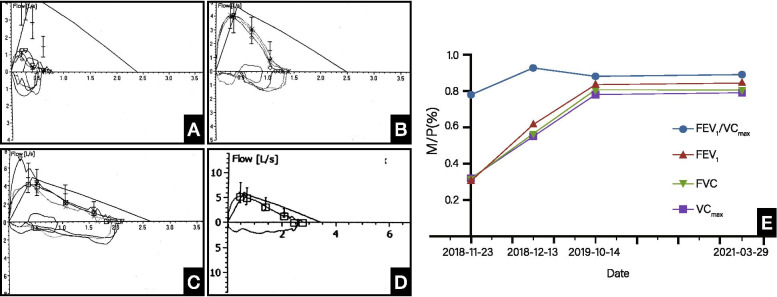
The follow-up of the lung function test for CAJDM-ILD. (**A**: Lung function test at admission, VC_MAX_, FVC, FEV_1_ and FEV_1_/VC_max_ were 0.79 L, 0.73 L, 0.61 L and 0.78, respectively, accounting for 32.2, 30.6, 30.7 and 91.9% of the predicted value; **B**: Lung function after one-month treatment, VC_MAX_, FVC, FEV_1_ and FEV_1_/VC_max_ were 1.39 L, 1.39 L, 1.29 L and 0.93, respectively, accounting for 54.6, 56.0, 62.0 and 109.4% of the predicted value; **C**: Lung function after one-year treatment, VC_MAX_, FVC, FEV_1_ and FEV_1_/VC_max_ were 2.11 L, 2.11 L, 1.86 L and 0.88, respectively, accounting for 78.0, 79.7, 84.0 and 104.1% of the predicted value; **D**: Lung function after 2.5-year treatment, VC_MAX_, FVC, FEV_1_ and FEV_1_/VC_max_ were 2.74 L, 2.74 L, 2.44 L and 0.89, respectively, accounting for 79.2, 79.8, 85.4 and 105.8% of the predicted value; **E**: Dynamic changes of VC_MAX_, FVC, FEV_1_, FEV_1_/VC_MAX_ in two and a half years)

This patient met the 2017 American college of rheumatology/European league against rheumatism classification criteria for adult and juvenile idiopathic inflammatory myopathies and was diagnosed with clinically amyopathic juvenile dermatomyositis (CAJDM) complicated by ILD. He received two courses of intravenous methylprednisolone pulse therapy (500 mg for 3 days a week) and intravenous immunoglobulin (IVIG) (1 g/kg for 2 days) followed by oral low-dose prednisolone (55 mg/d) and mycophenolate mofetil (MMF) (625 mg twice daily). Meanwhile, the patient received oxygen through the mask for 6 days and nasal oxygen for 19 days. After one month of treatment, his lung function test improved greatly (Fig. 3B) and his oxygen saturation reached 96%. We gradually reduced the prednisolone (30 mg/d) at the third-month treatment because his skin rash had improved (Fig. 2). There were no new muscle symptoms, dysphagia, calcinosis, arthralgia, joint contractors, lipodystrophy, lipoatrophy, or periungual capillary changes during the follow-up period. The patient was finally referred to the department of rheumatology to be followed up. CT performed one year after the initial treatment, and there was no exacerbation of abnormal findings. The lung function test at one-year follow-up presented mild restrictive dysfunction (Fig. 3C). The patient continued to be prescribed oral corticosteroid (5 mg/d) and mycophenolate mofetil (625 mg twice daily) after one year of treatment initiation for maintenance therapy. The last lung function test (Fig. 3D) showed that VCmax, FVC and FEV1 had significantly improved since the day of admission (Fig. 3E).

## Discussion and conclusion

To better understand the characteristics of this rare condition, we examined 7 reported cases of CAJDM-associated ILD for which good clinical records were available, including the present case [7–12]. All cases have confirmed the diagnosis of CAJDM based on the skin biopsy, and ILD was radiologically diagnosed. Clinical manifestations and course of illness for these 7 children with CAJDM-associated ILD are shown in Table 1.

**Table 1:** Clinical manifestations and course of illness in 7 children with CAJDM-associated ILD

PATIENT CHARACTERISTIC	CASE 1	CASE 2	CASE 3	CASE 4	CASE 5	CASE 6	CASE 7
AUTHOR (YEAR)	I. Kobayashi (2001)	I. Kobayashi (2003)	Yoshifusa Abe (2010)	Wang Tingjie (2015)	Jiang Lu (2017)	Our Case (2018)	Hou Jun (2019)
GENDER	Male	Male	Male	Female	Male	Male	Male
AGE AT ONSET Y	8	8	16	10	8	10	13
CUTANEOUS MANIFESTATIONS	Gottron papules	Gottron papules	Gottron papules	Gottron papules	Gottron papules Edema	Gottron papules	Gottron papules
MUSCLE MANIFESTATIONS	–	–	–	–	–	–	–
RESPIRATORY SYMPTOMS	Dry cough Dyspnea	Cough Dyspnoea	Cough Dyspnoea	–	–	Cough Chest pain	Chest pain
OTHER SYMPTOMS	Fever	–	–	Fever	Fever	Fever	–
WBC *10^9/L	3.9	Unknown	7.7	Unknown	Unknown	6.1	Normal
AST U/L	71	77	50	629	75	50	97
ALT U/L	38	Unknown	95	448	Normal	28	Unknown
CK U/L	1250	65	61	229	233	36	74
LDH U/L	647	539	392	Unknown	811	425	425
CRP MG/L	Negative	Unknown	0.3	Unknown	77	Negative	2.4
ESR MM/H	6	Unknown	Unknown	Normal	Normal	12	9
ANTI-MDA5	–	–	–	–	–	–	+
ANTI-RO52	–	–	–	+	–	+	–
OTHER ANTIBODIES	–	–	–	–	–	–	–
ELECTROMYOGRAPHY	Unknown	Unknown	Unknown	Myogenic damage	–	Normal	Unknown
CHEST CT	Interstitial pneumonia	Interstitial pneumonia	Interstitial pneumonia	Interstitial pneumonia	Interstitial pneumonia	Interstitial pneumonia	Interstitial pneumonia
LUNG FUNCTION	Unknown	Unknown	Unknown	Restrictive ventilatory dysfunction	Unknown	Restrictive ventilatory dysfunction	Unknown
SKIN BIOPSY	DM	Unknown	DM	DM	DM	DM	DM
TREATMENT	PSL AZA CSA	PSL AZA	MP CSA CTX	PSL IVIG PFD HCQ	MP IVIG MMF HCQ Tocilizumab	MP PSL MMF IVIG	MP CTX
PROGNOSIS	Died	Died	Died	Died	Improved	Improved	Not Improved

*WBC* white blood cell, *AST* aspartate aminotransferase, *ALT* alanine aminotransferase, *CK* creatine kinase, *LDH* lactate dehydrogenase, *CRP* C-reactive protein, *ESR* erythrocyte sedimentation rate, *chest CT* Chest computed tomography, *PSL* prednisolone, *AZA* azathioprine, *CSA* cyclosporin A, *MP* methylprednisolone, *CTX* cyclophosphamide, *IVIG* intravenous immunoglobulin, *PFD* pirfenidone, *MMF* mycophenolate mofetil, *HCQ* hydroxychloroquine

### Clinical manifestations

A male predominance was detected; this condition had a male: female ratio of 6:1. The mean age at onset of CAJDM-associated ILD is 10.4 years old, ranging from 8 to 16 years old.

Most of the patients (85.7%) began with skin manifestations (Gottron papules) as the initial presentation. However, the case we reported here presented respiratory symptoms (cough, dyspnea, chest pain) as its initial manifestation, which has not been reported before. His respiratory symptoms progressed after his skin rashes occurred. For the sake of early diagnosis, we focused on the possibility of linking initial respiratory symptoms with Gottron rashes to amyopathic dermatomyositis-associated ILD. To further testify this association, additional studies with larger patient samples from several centers are required for further investigations.

Interstitial lung disease (ILD) is a rarely reported complication of juvenile dermatomyositis [13, 14]. In all cases of adult DM-associated ILD, respiratory symptoms occur simultaneously or precede the skin or muscle manifestation [8, 15, 16]. Conversely, in most cases of CAJDM, respiratory symptoms were initially mild or absent, based on our report and literature review. As the asymptomatic pulmonary impairments of CAJDM-associated ILD worsen, especially the decrease in lung diffusion capacity, the prognosis become poor [17, 18]. Careful evaluation of pulmonary complications is necessary at the onset of CAJDM because prompt treatment can improve the prognosis. Recent studies have shown that CT findings of ILD are correlated highly to lung histopathological results, indicating that such findings may be a prompt and accurate means of assessing lung involvement in ILD at the early disease stage [19].

### Autoantibody profile

Anti-MDA-5 antibody was positive in one patient. Anti-Ro-52 antibody was weakly to moderately positive in two patients (28.6%). Other MSAs are negative in all the seven reported patients.

In the past few years, anti-MDA5 antibody has been proven to be a risk factor for developing ILD. It leads to poor outcomes in DM patients [3, 20, 21]. Recent cohort studies have shown that about half of anti-MDA5-positive adult CADM-associated ILD patients develop rapidly progressive ILD [22]. Anti-Ro52 antibodies are also common in systemic autoimmune diseases and may serve as an identifying element in the pathogenesis of this disease. Levels of these antibodies are significantly correlated with the development of ILD and cutaneous ulceration, and patients positive for anti-Ro52 antibodies are more likely to be refractory to the conventional immunosuppressive regimen, leading to a high mortality rate. In our review, one patient was positive for anti-MDA-5 antibody and two were weakly positive for anti-Ro-52 antibody (28.6%). Meanwhile, we identified four CAJDM-associated ILD patients in our study presenting double negative antibodies, three of whom died after a combined regimen of corticosteroids and immunosuppressants treatments. Previous works have established that anti-Ro52 antibody is highly common in anti-MDA5-positive CADM-ILD adults [3]. Conversely, no readily visible relationship between these two antibodies was found in our case review. Considering the limited simple size in our analysis, we believe further epidemiologic research in various age groups with CAJDM is warranted to understand the age-specific features of these two CAJDM-ILD-related antibodies.

### Treatment and prognosis

Corticosteroid therapy in the 1960s led to a drop in the mortality rate of ILD and a remarkable improvement in pulmonary outcomes [23]. However, ILD might still progress even during treatment with prednisolone and might become refractory to methylprednisolone pulse therapy. Later, advances in ILD disease management have been made by early aggressive intervention and the introduction of immunosuppressive medications with corticosteroid-sparing potential, leading to a robust decrease in disease-related mortality, which is currently < 2–3% [24].

Among the case series, four patients finally died of severe respiratory failure after taking corticosteroids and one or two immunosuppressive agents, including azathioprine (AZA), cyclosporin A (CSA), cyclophosphamide (CTX) and pirfenidone (PFD). One patient, used a regimen of corticosteroids and CTX, which is an established second-line treatment for severe or refractory JDM [25]. His skin manifestations improved, but his ILD progressively deteriorated until 2-year follow-up. CSA had a steroid-sparing effect on both skin and muscle lesions, supporting previous reports that CSA is effective in CAJDM-associated ILD [8]. Treatment with CTX appears to have major clinical benefits with no evidence of serious short-term toxicity in JDM patients. The skin and muscular and extra muscular features of the disease improved, and the improvement was maintained even after discontinuation of treatment [26]. However, in our review, there was little improvement in CAJDM-associated ILD patients who used CSA or CTX. More evidence is needed to determine their efficiency in the treatment of CAJDM-associated ILD.

Interestingly, the cutaneous and respiratory manifestations of other two patients who took IVIG together with corticosteroids and immunosuppressive drugs (MMF) were improved significantly. MMF is a lymphocyte inhibitor and it is well tolerated by teenagers, so it has become common in the treatment of autoimmune diseases. In our cohorts, we revealed that the addition of MMF and IVIG improved the skin rash and decreased the required dose of corticosteroid for maintenance therapy. This combined treatment can effectively control the active disease and induce remission in patients with CAJDM-associated ILD. More importantly, it can also avoid the need for high doses and prolonged therapy with corticosteroids, thereby bypassing severe long-term toxicity in young patients [27, 28]. Thus, we suggest future prospective controlled trials to further assess the use of MMF in CAJDM-associated ILD. Combined therapy of corticosteroids, immunosuppressants and IVIG may be a potentially effective means of controlling the course of ILD and may make a large contribution to better prognosis.

Respiratory symptoms could be the initial manifestations of interstitial lung disease in clinically amyopathic juvenile dermatomyositis. A combined regimen of corticosteroids, MMF and IVIG may potentially be a safer and more effective way to control disease and improve prognosis than current regimens.

## Data Availability

The datasets used and analysed during the current study are available from the corresponding author on reasonable request.

## Abbreviations

CAJDM: Clinically amyopathic juvenile dermatomyositis
JDM: Juvenile dermatomyositis
ILD: Interstitial lung disease
DM: Dermatomyositis
IIM: Idiopathic inflammatory myopathy
ALT: Alanine aminotransferase
AST: Aspartate aminotransferase
ESR: Erythrocyte sedimentation rate
CRP: C-reactive protein
CK: Creatine kinase
LDH: lactic dehydrogenase
Anti-MDA-5: Anti-melanoma differentiation-associated protein-5
MSAs: Myositis specific autoantibodies
MP-Ab: Mycoplasma pneumonia antibody
BALF: Bronchoalveolar lavage fluid
PAS: Periodic acid-Schiff stain
ANA: Antinuclear antibody
MRI: Magnetic resonance imaging
CT: Computed tomography
VC: Vital capacity
FVC: Forced vital capacity
FEV1: Forced expiratory flow in 1 s
CMAS: Childhood myositis assessment scale
EMG: Electromyography
PSL: Prednisolone
MP: Methylprednisolone
MMF: Mycophenolate mofetil
IVIG: Intravenous immunoglobulin
AZA: azathioprine
CSA: cyclosporin A
CTX: cyclophosphamide
PFD: Pirfenidone
HCQ: Hydroxychloroquine
